# Outpatient Cervical Ripening With Misoprostol in Low-Risk Pregnancies

**DOI:** 10.7759/cureus.19817

**Published:** 2021-11-22

**Authors:** Kristina Roloff, Kristina Nalbandyan, Suzanne Cao, C. Camille Okekpe, Inessa Dombrovsky, Guillermo J Valenzuela

**Affiliations:** 1 Department of Women's Health, Arrowhead Regional Medical Center, Colton, USA

**Keywords:** outpatient induction, misoprostol, outpatient cervical ripening, induction of labor, cytotec

## Abstract

Objective

To determine if outpatient cervical ripening with daily misoprostol can reduce admission to delivery time in women with low-risk pregnancies at 39 or more weeks of gestation.

Study design

This is a retrospective cohort study of a convenience sample of low-risk pregnancies that underwent elective outpatient cervical ripening compared to matched controls for parity (nulliparous vs. parous) and gestational age. Time from admission to delivery, induction agents, presence of tachysystole, mode of delivery, length of hospitalization, neonatal intensive care unit (NICU) admission, and low Apgar scores were compared.

Results

Fifty-six patients who underwent outpatient cervical ripening with daily dosing of misoprostol were compared to 56 patients matched for parity and gestational weeks who underwent inpatient cervical ripening/induction of labor with misoprostol. We found the time from admission to delivery in the outpatient cervical ripening cohort was significantly lesser than the inpatient cohort (17.5 ± 11.5 hours outpatient vs. 26.6 ± 15.6 hours inpatient, P=0.001). More patients (N=18, 32%) were able to deliver within 12 hours of admission in the outpatient induction group compared to the inpatient group (N=8, 11%, P=0.010). There were no differences in frequency of cesarean delivery, uterine tachysystole with or without fetal heart rate changes, NICU admission, low Apgar scores, or low umbilical artery pH values between the two groups.

Conclusion

Outpatient cervical ripening with misoprostol may be a feasible alternative to inpatient cervical ripening in low-risk pregnancies, may help improve patient experience, and reduce the operational burden that elective induction confers upon labor and delivery units.

## Introduction

The ARRIVE trial (A Randomized Trial of Induction Versus Expectant Management), amongst others, showed that the induction of labor in the 39th week of gestation may benefit both mother and baby alike, with reduced risks of cesarean delivery, hypertensive disorders of pregnancy, severe neonatal morbidity, and possibly stillbirth [1-3]. However, an increased rate of elective induction may place an operational burden on labor and delivery units [4,5]. Induction in women with less than optimal or “unripe” cervix may result in a prolonged inpatient stay, increased medical expense, and stress and exhaustion to the patient and her support people [6, 7]. Strategies such as outpatient cervical ripening may help to reduce the operational burden of elective induction but are under-studied [8-11].

Here, we review our experience with a pilot program of outpatient cervical ripening with daily misoprostol as an alternative to inpatient cervical ripening as part of the induction of labor in low-risk pregnancies. We hypothesized that outpatient cervical ripening with misoprostol would reduce hospitalized time to delivery.

## Materials and methods

This is a retrospective cohort study of women who underwent outpatient versus inpatient cervical ripening with misoprostol at a single institution (Arrowhead Regional Medical Center, ARMC) between 1 February 2017 and 31 March 2019. The primary outcome was to compare the length of time from admission to delivery in women who underwent outpatient cervical ripening with 25-50 µcg of misoprostol orally (PO) or per vagina (PV) daily, compared to those who received inpatient pre-induction cervix ripening with 25-50 µcg of misoprostol PO or PV every 4 to 6 hours with or without concomitant use of transcervical Foley catheter. As this was a pilot program, we wanted to ensure the primary aim of the protocol - to reduce time to delivery - was met. Secondary objectives were to determine any differences in tachysystole with or without fetal heart rate (FHR) changes, mode of delivery, complications, and neonatal intensive care unit (NICU) admission between outpatient and inpatient management. We defined tachysystole as six or more contractions in a 10-minute period.

Arrowhead Regional Medical Center is a 456-bed county teaching hospital that serves low socioeconomic status, publicly funded families residing in Southern California. ARMC performs approximately 2,500 deliveries per year. We began offering outpatient cervical ripening with daily misoprostol in February of 2017 in response to a noted increase in the frequency of elective induction after 39 weeks of gestation in optimally dated pregnancies, and an operational and physical burden to accommodate the influx of patients with prolonged hospitalization for induction. In addition, optimal and patient-preferred methods for outpatient cervical ripening are not known [10]. Attending physicians were wary of offering outpatient cervical ripening with a transcervical catheter due to concern that patients would not return timely for evaluation, as compliance with return visits was known to be poor. Hence, we decided to offer daily outpatient misoprostol instead.

Women were offered outpatient cervical ripening if the following criteria were met: (1) cephalic fetal position by ultrasound, (2) category 1 fetal heart rate tracing, (3) estimated fetal weight by physical examination or ultrasound under 4000 g, (4) estimated gestational age 39 weeks and optimally dated (confirmed by 22-week or earlier ultrasound), (5) low-risk pregnancy (diet dependent diabetes, advanced maternal age, and obesity with BMI <45 were allowed), (6) maternal report of adequate fetal movements, (7) amniotic fluid between 2 and 8 cm as documented by maximum vertical pocket method on ultrasound, (8) modified Bishop score ≤ 7, (9) adequate transportation to and from the hospital and staying less than 30 minutes from our institution, and (10) agrees to outpatient induction. Women with medical, obstetric, or gynecologic comorbidities (e.g., hypertensive disorder, pre-gestational diabetes, insulin-dependent diabetes, seizure disorder, lupus, etc.), fetal malformations, fetal growth restriction, disorders of amniotic fluid, and women with a prior cesarean delivery or uterine surgery were not offered outpatient induction.

The dose of misoprostol (25 or 50 µcg) and route (PO or PV) was left to the attending physician’s discretion, and either dose could be given PO or PV. Women underwent 120 minutes of continuous fetal heart rate monitoring and tocometry following misoprostol administration, based on known misoprostol peak plasma levels of misoprostol (60 minutes after a single vaginal dose, and approximately 30 minutes following oral administration) [12]. Administration of misoprostol and fetal heart rate monitoring was performed on the labor and delivery unit, and observed and interpreted by a labor and delivery nurse. If the fetal heart rate remained reassuring, and tocometry and the patient reported feeling fetal movements and no symptoms of strong uterine activity, they were discharged home with instructions to return in 24 hours for re-evaluation. The once-a-day misoprostol administration was chosen for the convenience of day-time application and night-time rest for patients and providers alike.

Daily evaluation of women undergoing outpatient cervical ripening consisted of fetal heart rate monitoring and amniotic fluid measurement, physical examination including cervix examination and assessment of the presence of contractions, adequate fetal movements, and maternal desire to continue outpatient cervical ripening. Misoprostol was administered q 24 hours for up to four doses. The patient was instructed to return immediately to the labor and delivery unit for vaginal bleeding or spotting, frequent painful contractions, leakage of fluid, or decreased fetal movements. Women were admitted for inpatient management if they presented with any of these complaints, or at any time for maternal request for inpatient management, or on day #4 - considered completion of the outpatient protocol. 

Controls were recruited from the inductions performed closest in time to the outpatient case to control for progressive changes in the practice. Controls were matched for gestational weeks and for parity (nulliparous or parous). Inductions performed without the use of misoprostol for cervical ripening were excluded to limit the confounding effects of different induction modalities. Typical inpatient cervix ripening at our facility is with 25 or 50 µcg misoprostol every 4 hours for 1-4 doses, with or without the use of a transcervical Foley catheter. As this was a pilot project, we did not have a priori data on length of hospitalization time to delivery to determine sample size and selected an arbitrary sample size of 50 in each cohort.

The following variables were abstracted from retrospective chart review of cases and controls: age, gravidity, parity, ethnicity, gestational age, body mass index (BMI kg/m2), indication for admission if outpatient cervical ripening started, modified Bishop score at the start of induction, modified Bishop score at hospital admission, presence of tachysystole with or without fetal heart rate changes during hospitalization, time of hospital admission, time of delivery, mode of delivery, indication for cesarean, maternal or neonatal complications, neonatal Apgar scores, and neonatal umbilical cord arterial and venous blood gas results.

Data was summarized by standard descriptive summaries (mean and standard deviation for continuous variables and number and percentage for categorical variables). Statistical comparison to gestational age and parity matched controls was performed using a 2-tailed t-test, chi-squared test, or Fischer’s exact test where appropriate for ordinal or categorical variables. An intent to treat analysis was performed with non-compliant patients and those with fetal heart rate changes or labor following first misoprostol administration included in the outpatient cohort, to better reflect the actual experience and results of the pilot program. A planned sub-analysis excluded patients non-compliant with follow-up, or immediate labor or fetal heart rate changes following misoprostol administration, in order to isolate the effect of outpatient once-daily misoprostol use on hospitalization time to delivery in women who underwent planned cervical ripening at home. A planned sub-analysis was also performed for all patients who achieved vaginal delivery to exclude bias from women who had prolonged admission to delivery times due to failure to progress, and those who had deliveries interrupted due to concerns over fetal heart rate tracing changes. The women who presented for scheduled outpatient induction but were not considered candidates at that time (already in labor, contracting too frequently for misoprostol administration) were excluded from the analyses. Statistical analysis was conducted with SPSS version 23.0.0.0 (IBM, Armonk, NY, USA). A two-sided p<0.05 was considered statistically significant. This study was conducted with the approval of the ARMC Institutional Review Board.

## Results

**Figure 1:**
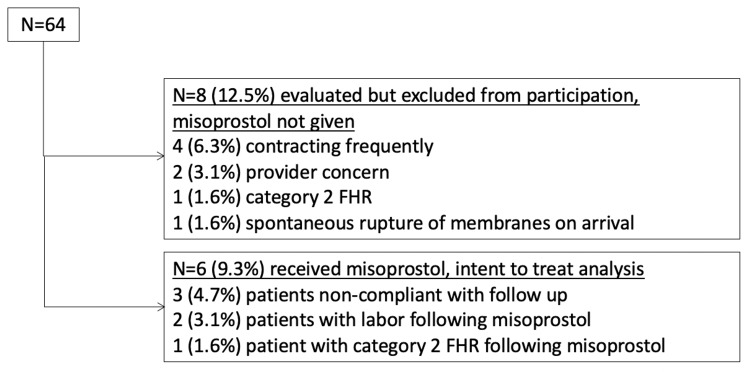
Reasons for participation or exclusion in outpatient induction. FHR, fetal heart rate

Fifty-six patients elected outpatient induction during the study period. Six (10.7%) patients started the outpatient induction protocol but did not complete or comply with the protocol. Three received misoprostol but did not return daily as instructed but ultimately returned and delivered, two had an immediate onset of labor following the first dose of misoprostol, and one had a category 2 FHR pattern following the first dose of misoprostol and so was kept for inpatient induction (Figure 1).

Demographic and admission characteristics of the 56 patients in each cohort are presented in Table 1. There were no significant differences noted between the two groups.

**Table 1:** Demographic characteristics of patients in the outpatient and inpatient induction cohorts. Data are N (%) or mean ± standard deviation.

	Outpatient induction (N=56)		Inpatient induction (N=56)		P
Age	23.7	±3.8	24.87	±4.9	0.181
Gravidity	2.07	±1.3	1.93	±1.2	0.398
Parity	0.71	±0.9	0.73	±1.1	0.426
Gestational age	39.5	±0.6	39.5	±0.5	0.656
Ethnicity					
Hispanic	48	(85.7)	44	(78.6)	0.382
Black	5	(8.9)	8	(14.4)	
White	3	(5.4)	2	(3.6)	
Asian	0	(0.0)	2	(3.6)	
BMI	32.2	±6.0	34.3	±7.2	0.134
Modified Bishop Score	1.31	±1.4	1.56	±1.6	0.292
Dilation (cm)	0.57	±0.8	0.95	±0.9	0.212
Effacement %	16.9	±25.6	33.0	±28.0	0.329
Station (-5 to +5)	-3.4	±1.3	-3.4	±1.4	0.420

We found the time from admission to delivery in the outpatient cervical ripening cohort was significantly less than compared to the inpatient cohort (17.5 ± 11.5 hours outpatient, vs. 26.6 ± 15.6 hours inpatient, P=0.001, statistical power 94%). More patients (N=18, 32%) were able to deliver within 12 hours of admission in the outpatient induction group compared to the inpatient group (N=8,11%, P=0.010). Of patients who were compliant with the study protocol and were discharged for outpatient cervical ripening from the outpatient cohort, one patient (2%) was admitted on day 1, 22 (39%) were admitted on day 2, 8 (14%) were admitted on day 3, and the remaining 19 (34%) completed the four-day protocol.

A sub-analysis of 50 women and 50 matched controls, which excluded the six women who did not comply with the protocol, or had an immediate onset of labor or fetal heart rate changes that precluded them from going home for cervical ripening, was performed. Results showed similar demographic and physical characteristics between the inpatient and outpatient groups, except for initial cervix dilation: women in the outpatient cohort were slightly less dilated at the start of induction than in the inpatient cohort (0.54 ±0.7 cm, 0.94 ±1.0 cm, P=0.046). Like the intent-to-treat analysis above, we found the time from admission to delivery in the outpatient cervical ripening group was significantly less than compared the inpatient group (17.7 ± 11.0 hours outpatient, vs. 27.9 ± 16.0 hours inpatient, P=0.001). More patients (N=16, 32%) were able to deliver within 12 hours of admission in the outpatient induction group compared to the inpatient group (N=6,12%, P=0.029).

In a sub-analysis of women who achieved vaginal delivery (N=89, 79%), the time from admission to delivery in the outpatient group was significantly less than the inpatient group (16.2 ±12.1 hours outpatient, vs. 23.8 ±11.7 hours inpatient, P=0.007, statistical power 82%). More women delivered within 12 hours of admission in the outpatient group (N=16, 34% outpatient, N=6, 14% inpatient, P=0.030).

We found no significant differences between the inpatient and outpatient cohorts in our intention-to-treat analysis, analysis of women who were compliant and were discharged for at-home cervical ripening at least once, or sub-analysis of those who achieved vaginal birth for birthweight, epidural use, NICU admissions, low Apgar scores, or low umbilical artery pH values. Notably, the chances of both tachysystole with and without fetal heart rate changes during hospitalization were comparable in both the inpatient and outpatient cohorts. The most common complication was chorioamnionitis, which occurred in 7.1% of all patients, and more often in the outpatient cohort (N=6, 10.7% outpatient, vs. N=2, 3.6% inpatient, P=0.271), though this did not reach statistical significance. All patients that developed chorioamnionitis in the outpatient cohort were admitted on day 4 of the protocol. One patient had a rectovaginal Group B Streptococcus (GBS) culture that was positive, and one infant developed GBS septicemia despite a negative 36-week rectovaginal culture in the mother. Postpartum hemorrhage occurred in more of the inpatient cervical ripening cohort compared to the outpatient cohort, but this was not statistically significant (N=1, 1.8%, vs. outpatient, N=5, 8.9% inpatient, P=0.206). Overall, complications were not different between the groups, but the frequency of complications was low, and the study was not powered to find small differences. Delivery characteristics and complications are shown in Table 2.

**Table 2:** Delivery characteristics of outpatient and inpatient induction of labor. Data are N (%) or mean ±standard deviation. NICU, neonatal intensive care unit

	Outpatient induction		Inpatient induction		P
Birthweight	3401	±319.8	3323	358.6	0.501
Route of delivery					
Spontaneous vaginal	47	(83.9%)	39	(69.6%)	0.089
Vacuum assisted vaginal	0	(0.0%)	3	(5.4%)	
Cesarean delivery	9	(16.1%)	14	(25.0%)	
Complications					
Postpartum hemorrhage	1	(1.8%)	5	(8.9%)	0.206
Uterine atony	0	(0.0%)	1	(1.8%)	N/A
Chorioamnionitis	6	(10.7%)	2	(3.6%)	0.271
Precipitous delivery	1	(1.8%)	0	(0.0%)	N/A
Unstable lie	1	(1.8%)	0	(0.0%)	N/A
Shoulder dystocia	1	(1.8%)	0	(0.0%)	N/A
Severe preeclampsia	1	(1.8%)	0	(0.0%)	N/A
Epidural use	48	(85.7%)	48	(85.7%)	1.000
NICU admission	1	(1.8%)	1	(1.8%)	1.000
Apgar 1 minute ≤ 5	1	(1.8%)	3	(5.4%)	0.618
Umbilical artery pH <7	0	(0.0%)	1	(1.8%)	N/A
Tachysystole	23	(41.0%)	18	(32.0%)	0.416
Tachysystole + fetal heart rate changes	5	(8.9%)	3	(5.4%)	0.715
Admission to delivery time (hours)					
Delivery <12 hours	16	(29%)	6	(11%)	0.029

All but two patients received 50 µcg of misoprostol by oral route in the outpatient cervical ripening cohort, and two (3.6%) received a combination of vaginal and oral misoprostol. Forty-eight (85.7%) patients were given oral misoprostol in the inpatient cohort, at a slightly lower mean dose than compared to the outpatient cohort (50 µcg outpatient, 49.5 ±3.61 µcg inpatient, P=0.04). The total number of misoprostol doses given in the outpatient cervical ripening cohort was significantly higher (2.46 ±1.14, outpatient, vs 1.98 ±0.98, inpatient, P=0.032). Additional induction agents and oxytocin use are shown in Table 3. Fifty-five (98%) of patients in the inpatient cohort received agents in addition to the initial misoprostol dose, compared to 51 (91%) patients in the outpatient cohort. There was no significant difference in the number of cases exposed to oxytocin, or in the maximum oxytocin dose used between the two groups.

**Table 3:** Additional induction agents and oxytocin use in women undergoing outpatient compared to inpatient induction of labor. Data are N (%) or mean ±standard deviation.

	Outpatient induction			Inpatient induction			P	
Additional agents in cervical ripening	51	(91%)	55		(98%)	0.056		
Foley + misoprostol	1	(2%)	0		(0%)	0.522		
Foley + misoprostol + oxytocin	25	(49%)	26		(47%)			
Dinoprostone	0	(0%)	1		(2%)			
Oxytocin	23	(45%)	28		(51%)			
Oxytocin used	50	(89%)	55		(98%)	0.113		
Maximum oxytocin dose if used (milliunits/minute)	11.36	±7.55	11.42		±8.07	0.986		

The primary cesarean delivery rate for the entire sample was 21% (N=23), and there was no difference in the mode of delivery between the inpatient and outpatient cohorts. The most common indication for cesarean delivery was the failure to progress (N=9, 39%), followed by fetal heart rate concerns (N=8, 35%). The remaining cesarean deliveries were for malpresentation in a patient with an unstable lie, failed vacuum extraction, chorioamnionitis, and failed induction.

## Discussion

Principle findings

We found a marked (~9-10 hour) reduction in length of time from admission to delivery in women who underwent outpatient cervical ripening with daily misoprostol, compared to controls matched for parity and gestational age who underwent inpatient cervical ripening with misoprostol as part of an induction of labor, without any difference in frequency of uterine tachysystole with or without fetal heart rate changes, NICU admission and cesarean delivery.

Results

Several published studies of outpatient misoprostol for cervical ripening compared to expectant management or placebo suggest a reduction in time to the active phase of labor without significant increase in side effects [13-18]. To our knowledge, this is the first study to systematically compare outpatient cervical ripening to inpatient induction with misoprostol. 

Outpatient cervical ripening using a transcervical catheter has been proposed as a safe and reasonable alternative to inpatient induction to alleviate resource allocation as the number of induced women increases in light of evidence of benefit [9, 19-21]. However, it is unclear if outpatient transcervical Foley will reduce operational burden. Studies on outpatient pre-induction cervical ripening with a transcervical Foley catheter show conflicting results regarding admission to delivery interval [19, 20, 22-25]. In addition, outpatient transcervical Foley alone for cervical ripening may not improve time to delivery compared to inpatient combined transcervical Foley with oxytocin [23]. As in our practice, providers may be concerned about compliance with return visits and reluctant to implement an outpatient transcervical Foley catheter protocol. Here, we pilot an alternative option for outpatient cervical ripening.

Clinical implications

As we increase the number of elective inductions at 39 weeks in light of evidence of benefit, strategies to decrease operational and physical factors on labor and delivery units are needed to facilitate optimal delivery timing.

Larger studies are needed to understand the safety profile of outpatient cervical ripening with misoprostol, but we have shown here that there may be some benefit in terms of reduction of time from admission to delivery. We found more cases of chorioamnionitis in women undergoing outpatient cervical ripening, but an overall very low chance of chorioamnionitis (7.1% in the entire cohort), and fewer cases overall when compared to women who underwent induction of labor in the ARRIVE trial (13.3%) [1]. Our rates of chorioamnionitis were comparable to other studies on induction of labor with misoprostol (7.5-8%) [26,27]. Most cases of chorioamnionitis were in women who received all four doses of misoprostol in the outpatient setting, suggesting the outpatient protocol may need to be shortened to 3 days, or that we should identify strategies to find misoprostol ‘non-responders’ prior to cervix ripening. Since two cases of neonatal GBS infection were found in women with clinical chorioamnionitis, we may need to consider the safety of outpatient induction in women with rectovaginal GBS carriage at 36-37 weeks.

Research implications

A survey of women in the antenatal period shows interest and willingness to participate in outpatient cervical ripening [28]. Outpatient management also may be more preferable to some women who may feel more comfortable at home and could encourage greater movement and hence physiology of the normal labor process [8, 10]. However, there is little published on patient satisfaction or interest in outpatient cervical ripening, and this is an area where more research is clearly needed.

Strengths and limitations

The strength of this study is its pragmatic and timely nature. However, the study sample size is small. This study was not powered to determine differences in maternal or neonatal complications due to their scarcity, and further research is needed to determine any hazard of outpatient cervical ripening with misoprostol.

## Conclusions

Outpatient cervical ripening with misoprostol may be a feasible alternative to inpatient cervical ripening in low-risk pregnancies and may help improve patient experience, and reduce the operational burden that elective induction confers upon labor and delivery units.
